# Z-Scheme BiVO_4_/g-C_3_N_4_ Photocatalyst—With or Without an Electron Mediator?

**DOI:** 10.3390/molecules29215092

**Published:** 2024-10-28

**Authors:** Tomasz Łęcki, Kamila Zarębska, Ewelina Wierzyńska, Krzysztof P. Korona, Paulina Chyży, Piotr Piotrowski, Magdalena Skompska

**Affiliations:** 1 Faculty of Chemistry, University of Warsaw, Pasteur 1, 02-093 Warsaw, Poland; t.lecki@uw.edu.pl (T.Ł.); kzarebska@chem.uw.edu.pl (K.Z.); em.kwiatkowska@uw.edu.pl (E.W.); pe.chyzy@student.uw.edu.pl (P.C.); ppiotrowski@chem.uw.edu.pl (P.P.); 2 Biological and Chemical Research Centre, University of Warsaw, Żwirki i Wigury 101, 02-089 Warsaw, Poland; 3 Faculty of Physics, University of Warsaw, Pasteur 5, 02-093 Warsaw, Poland; krzysztof.korona@fuw.edu.pl

**Keywords:** photocatalysis, composite materials, Z-scheme, pollutant degradation, IMPS, TPRL

## Abstract

The hybrid system BiVO_4_/g-C_3_N_4_ is a prospective photocatalyst because of the favorable mutual alignment of the energy bands of both semiconductors. However, the path of the photocatalytic process is still unclear because of contradictory information in the literature on whether the mechanism of charge carrier separation at the BiVO_4_/g-C_3_N_4_ interface is band-to-band or Z-scheme. In this work, we clarified this issue by comparative photocatalytic studies with the use of systems without a mediator and with different kinds of mediators including Au nanoparticles, fullerene derivatives, and the Fe^3+^/Fe^2+^ redox couple. Additionally, the charge transfer dynamics at the BiVO_4_/g-C_3_N_4_ and BiVO_4_/mediator/g-C_3_N_4_ interfaces were investigated by time-resolved photoluminescence (TRPL) measurements, while the influence of the mediator on the surface recombination of the charge carriers was verified by intensity-modulated photocurrent spectroscopy (IMPS). We proved that the charge carrier separation at the BiVO_4_/g-C_3_N_4_ interface occurs according to the mechanism typical for a heterojunction of type II, while the incorporation of the mediator between BiVO_4_ and g-C_3_N_4_ leads to the Z-scheme mechanism. Moreover, a very strong synergetic effect on caffeine (CAF) degradation rate was found for the system BiVO_4_/Au/g-C_3_N_4_ in the presence of Fe^3+^ ions in the CAF solution.

## 1. Introduction

The development of efficient, stable, cheap, and environmentally friendly systems for solar-to-chemical energy conversion (water splitting, CO_2_ reduction, organic pollutant degradation, etc.) is still a hotspot in the field of photocatalysis. To achieve these goals, photocatalytic systems should meet several requirements, such as photoactivity under visible light, a low recombination rate of photogenerated charge carriers, and a high oxidation and reduction power of the holes and electrons, respectively. All these demands cannot be fulfilled by a single semiconductor; therefore, hybrid systems composed of two semiconductors with appropriate mutual alignment of the conduction band (CB) and valence band (VB) edges are proposed. The most suitable are those with one semiconductor (SC I) effective in oxidation and the second one (SC II) in reduction. It is also expected that these hybrid systems operate according to the Z-scheme, i.e., the electrons photogenerated in SC I recombine directly or via a mediator with the holes from the VB of SC II. Then, the electrons from the CB of SC II, with stronger reduction ability, and the holes from the VB of SC I, with high oxidation potential, can be involved in the redox reactions with the solution species [1,2]. Taking into account the activity of photocatalysts in visible light, the most attractive are the systems such as WO_3_/g-C_3_N_4_ [3,4], WO_3_/CdS [5], Ag_3_PO_4_/g-C_3_N_4_ [6,7], BiVO_4_/g-C_3_N_4_ [8,9], or α-Fe_2_O_3_/Cu_2_O [10]. An important question still discussed in the literature is whether the mechanism of separation of the charge carriers in these hybrid systems is band-to-band transfer, direct Z-scheme, or indirect (mediated) Z-scheme. For example, in the case of BiVO_4_/g-C_3_N_4_, the formation of a heterojunction of type II between these two components has been postulated by several groups [11,12,13,14]. According to other reports, BiVO_4_ coupled with g-C_3_N_4_ forms a direct Z-scheme photocatalyst, owing to strong interactions between the facets (002) of g-C_3_N_4_ and (121) of BiVO_4_ [9] or tight face-to-face interfacial contact between these two materials prepared in the form of 2D nanosheets [15]. In contrast, several types of solid mediators, such as Au [16], Ag [17], tetragonal BiVO_4_ [18], carbonaceous materials, such as RGO (reduced graphene oxide) [19,20], carbon nanotubes (CNTs) [21], and the redox Fe^3+^/Fe^2+^ couple [22], have been used to achieve a good performance of the BiVO_4_/g-C_3_N_4_ photocatalytic system. However, it is very difficult to compare all these literature data because the reported hybrid systems have been fabricated by different methods, such as wet impregnation [21], hydrothermal synthesis [14,19], the mixed calcination method [9], and drop casting of g-C_3_N_4_ on the surface of BiVO_4_ [8]. Moreover, they were applied in different photocatalytic reactions including hydrogen evolution [16,17], water oxidation [18], CO_2_ conversion [15], degradation of dyes [9,14], antibiotics [19], and phenol [21].

Therefore, the aim of this work was to clarify the mechanism of the electron–hole separation in the irradiated BiVO_4_/g-C_3_N_4_ system and verify the need for a mediator to obtain the Z-scheme photocatalyst. In order to achieve these goals, we performed systematic investigations of binary and ternary composites containing BiVO_4_, g-C_3_N_4_, and various mediators, including Au nanoparticles (Au NPs), fullerene derivatives, and the redox couple Fe^3+^/Fe^2+^ in the solution, to determine step by step the role of each component in the photocatalytic process. According to the literature, fullerenes can be used as electron acceptors and donors [23], but, to the best of our knowledge, this is the first report on their application as charge transfer mediators in the Z-scheme system. To obtain a reliable comparison, BiVO_4_ and g-C_3_N_4_ were prepared using very reproducible procedures, and their amounts deposited on the solid substrate were controlled. BiVO_4_ was fabricated on FTO by means of an electrochemical/chemical method to ensure very good electrical contact with the conducting substrate, which is crucial in electrochemical measurements. The exfoliated flakes of g-C_3_N_4_ were applied on BiVO_4_ by drop casting. The role of the mediators was determined by taking into account the rate of photocatalytic degradation of caffeine (CAF) at the surface of the prepared hybrid systems, while the dynamics of charge transfer and recombination processes were investigated by means of IMPS (intensity-modulated photocurrent spectroscopy), photoluminescence, and time-resolved photoluminescence (TRPL) measurements.

## 2. Results

### 2.1. Morphology and Optical Properties of BiVO_4_, BiVO_4_/Au(NPs), BiVO_4_/Fullerene, and BiVO_4_/Au(NPs)/g-C_3_N_4_

**Figure 1:**
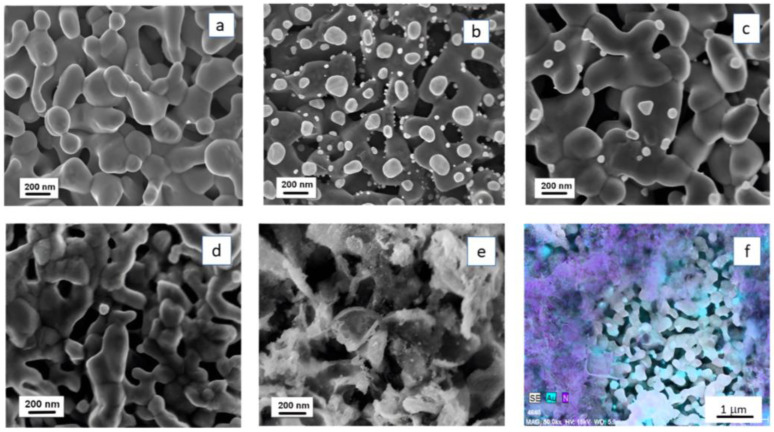
SEM images of (**a**) BiVO_4_, (**b**) BiVO_4_/Au NPs obtained by Au sputtering and (**c**) by drop casting of HAuCl_4_, followed by annealing, (**d**) BiVO_4_/C_60_-MPB, (**e**) BiVO_4_/Au(NPs)/g-C_3_N_4_. (**f**) Distribution of N and Au in BiVO_4_/Au(NPs)/g-C_3_N_4_ for selected part of the sample.

The SEM images of bare BiVO_4_ and BiVO_4_ decorated with Au NPs by two different methods, sputtering (Au-s) and drop casting (Au-d) (see details in Section 3.3), are presented in Figure 1a–c. After Au sputtering, the surface of worm-like BiVO_4_ (Figure 1a) was covered with a uniform layer of Au (Figure S1a). The annealing of the sample led to the conversion of the Au layer into Au NPs with a very large size distribution, from 20 nm to about 300 nm (Figure 1b). Deposition of Au by drop casting of HAuCl_4_, followed by annealing of the sample, resulted in the formation of NPs of different shapes (triangles, spheres, and cubes) with quite a small size distribution (from 30 to 90 nm) (Figure 1c). The analysis of EDX spectra showed that the relative amount (in at %) of Au: Bi(or V) in the samples prepared by Au sputtering was more than two times higher than that in the samples obtained by drop casting (Table 1).

The initial photocatalytic experiments indicated that the system BiVO_4_ /(Au-s) revealed lower photocatalytic activity than BiVO_4_ /(Au-d), because in the former case, a relatively large surface area of BiVO_4_ was screened by Au NPs, and the number of photogenerated charge carriers was probably much lower than that in the sample BiVO_4_/(Au-d). Thus, the drop casting method was selected for the preparation of Au NPs in the ternary BiVO_4_/Au/g-C_3_N_4_ systems.

The presence of fullerene derivatives on the surface of BiVO_4_ is manifested in the SEM images by darker zones (Figure 1d), because of the nanometer size of C_60_ and C_70_ cages. After application of g-C_3_N_4_, exfoliated in DMSO into nanometer-thick lamellas (Figure S1b), the polymer formed the “seaweed”-like areas of high porosity on the surface of BiVO_4_ (Figure 1e). This provides a good contact of both components with the solution species, and also a direct contact between g-C_3_N_4_ and BiVO_4_, which is essential for efficient charge transfer between these two semiconductors. The elemental mapping of the sample BiVO_4_/Au(NPs)/g-C_3_N_4_ showed that some parts of BiVO_4_ were not covered with g-C_3_N_4_ (Figure 1f). Analysis of the EDX spectrum of the sample (Figure S2) indicated that the atomic ratio C:N is 0.77, which is very close to the theoretical value of 0.75 for g-C_3_N_4_ and the atomic ratio V:B (0.97) also meets the value expected for BiVO_4_ (see results in Table 1).

**Figure 2:**
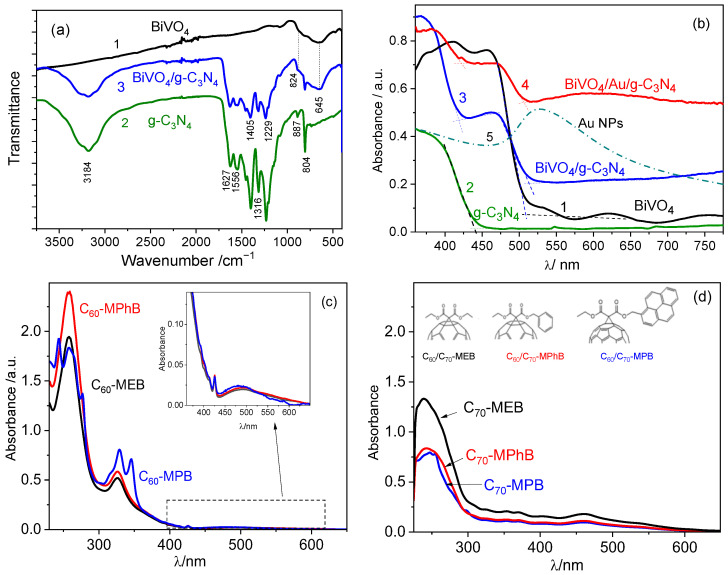
(**a**) FTIR spectra and (**b**) optical absorption spectra of BiVO_4_ (lines 1), g-C_3_N_4_ (lines 2), BiVO_4_/g-C_3_N_4_ (lines 3), BiVO_4_/Au(NPs)/g-C_3_N_4_ (line 4), and Au(NPs) suspended in aqueous solution (line 5). UV-Vis absorption spectra of fullerene derivatives: (**c**) C_60_-MEB, C_60_-MPB, and C_60_-MPhB, and (**d**) C_70_-MEB, C_70_-MPB, and C_70_-MPhB dissolved in dichloromethane (chemical structures of fullerene derivatives are depicted in the inset).

In the FTIR spectra of the hybrid system BiVO_4_/g-C_3_N_4_ presented in Figure 2a, there are bands characteristic of both components. A broad band with a maximum at 645 cm^−1^ and a shoulder at about 830 cm^−1^ originate, respectively, from the bending vibration of Bi-O and stretching vibration of V-O bonds in BiVO_4_ [24], while five dominant peaks in the range from 1200 cm^−1^ to 1627 cm^−1^ correspond to stretching vibrations of C-N and C=N bonds in heterocycles in g-C_3_N_4_ [25]. A sharp peak at approximately 804 cm^−1^ is attributed to a characteristic breathing mode of tri-s-triazine cycles (ring stretching vibrations) [26].

Both components of the hybrid system absorb photons in the visible range, as illustrated in Figure 2b (lines 1 and 2). The absorption spectrum of the binary BiVO_4_/g-C_3_N_4_ system (line 3) is a combination of the spectra of both components since BiVO_4_ film is not completely covered with g-C_3_N_4_. The band gap energies of BiVO_4_ and g-C_3_N_4_ were determined from Tauc’s plots (based on the Kubelka–Munk function) for direct and indirect semiconductors, respectively (Figure S3). The obtained values (2.48 eV for BiVO_4_ and 2.82 eV for g-C_3_N_4_) are in very good agreement with the literature data [27,28]. It was also interesting to note that practically the same values of the band gaps (2.43 eV for BiVO_4_ and 2.82 eV for g-C_3_N_4_) were obtained directly from the edges of absorption bands in UV-Vis spectra (observed at the wavelengths 514 nm and 440 nm, respectively) with the use of the relationship E(eV) = 1240/λ(nm).

In the optical spectrum of the ternary system BiVO_4_/Au(NPs)/g-C_3_N_4_ (line 4 in Figure 2b), one can observe an additional absorption band with a maximum at about 590 nm, ascribed to a plasmonic band of Au nanoparticles. The position of the band maximum is red-shifted with respect to the position of this peak for nanoparticles suspended in the solution (about 525 nm, line 5), which can be explained by a different dielectric constant of the environment surrounding the nanoparticles and interactions of Au NPs with semiconductor [29] . Thus, under illumination of the photocatalytic systems containing Au mediator with a diode at 400 nm, the plasmon resonance effect in which the electrons are transferred from the excited plasmonic state to the CB of BiVO_4_ may be neglected, and therefore, the role of Au NPs as the electron mediator can be clearly determined.

As visible in Figure 2c,d, the C_60_ and C_70_ fullerene derivatives used in this work (depicted in the inset in Figure 2d) absorb photons in the UV range, while absorption of the visible light is negligible, and therefore, the presence of fullerenes has no effect on the spectrum of the hybrid system.

### 2.2. Photocatalytic Activity of BiVO_4_, BiVO_4_/Au, BiVO_4_/Fullerene and BiVO_4_/Fe^3+^

**Figure 3:**
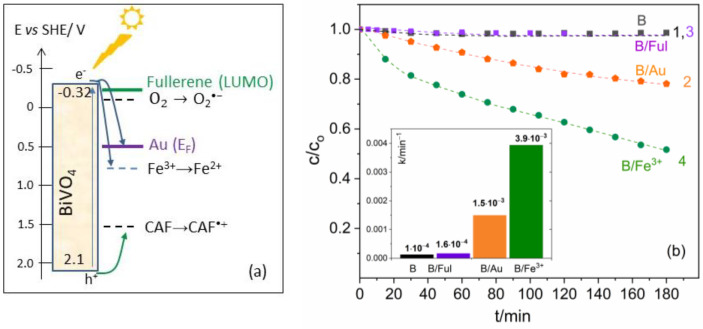
(**a**) Band diagram of BiVO_4_ with indicated potentials corresponding to the Fermi level of Au, LUMO level of the fullerenes, and oxidation/reduction potentials of the solution species involved in the photocatalytic process. (**b**) Changes i the concentration ratio (c/c_o_) of CAF in a function of irradiation time with a diode 400 nm, in the presence of bare BiVO_4_ (B, line 1), BiVO_4_/Au(NPs) (B/Au, line 2), BiVO_4_/fullerene (B/Ful, line 3), and BiVO_4_ in the presence of Fe^3+^ ions in the CAF solution (B/Fe^3+^, line 4); inset: the corresponding rate constants of CAF degradation.

In the first stage of investigations, BiVO_4_ was combined with Au NPs and fullerenes, which were later used as the charge transfer mediators in the ternary systems, to determine their influence on the photocatalytic activity of BiVO_4_ in the degradation of caffeine (CAF). According to our previous photoelectrochemical studies [30], the bottom edge of the CB in BiVO_4_ is located at about −0.32 V vs. SHE, while the upper edge of the VB is situated at the potential of 2.1 V vs. SHE, as depicted in Figure 3a. Since the onset potential of CAF oxidation determined from the cyclic voltammogram is 1.53 V vs. SHE (Figure S4a), the holes photogenerated in BiVO_4_ can be involved in direct oxidation of CAF (see Scheme S1). In contrast, the holes are not able to oxidize water molecules to hydroxyl radicals •OH, because the standard potential of •$OH/H_{2}O$ (2.3 V vs. SHE at pH 7 [31]) is more positive than the E_VB_ of BiVO_4_ [30,32,33]. However, the hydroxyl radicals, which are the second oxidizing agent in CAF degradation (Scheme S2), may be formed by the transformation of superoxide anion radicals ($O_{2}^{⦁-}$) according to the scheme [34]:

(1) $$O_{2}^{•-}+H^{+}⇌{HO}_{2}^{•}$$

(2) $${HO}_{2}^{•}+e^{-}+H^{+}\to H_{2}O_{2}$$

(3) $$H_{2}O_{2}+e^{-}\to {OH}^{-}+•OH$$

The superoxide radicals may be created since the bottom edge of the CB is located above the redox potential of $O_{2}/O_{2}^{⦁-}$ couple (−0.18 V vs. SHE, in aqueous solution [35]), as illustrated in the band diagram in Figure 3a, but the difference between these two potentials is rather small (0.14 V). Although the band gap energy of BiVO_4_ is lower than the energy of photons emitted from the diode used in the experiments (3.1 eV), the photocatalytic activity of bare BiVO_4_ was very low (line 1 in Figure 3b), which is explained by a slow charge carrier transport [36] and fast electron–hole (*e^−^–h^+^*) recombination [37]. As visible in Figure 3b, decoration of BiVO_4_ with Au NPs (the system abbreviated as B/Au) led to an evident decrease in CAF concentration (line 2) under illumination of the system with the same diode (the UV-Vis spectra recorded during the photocatalytic process are presented in Figure S5a). Since the surface plasmon resonance (SPR) of Au NPs occurs at the wavelength of 590 nm, the observed photocatalytic improvement may be ascribed to hindered recombination of the charge carriers, owing to transfer of photoinduced electrons from BiVO_4_ to Au NPs. Then, the holes from the VB of BiVO_4_ can oxidize CAF.

In contrast to Au NPs, the application of fullerene on the surface of BiVO_4_ did not improve the photocatalytic decomposition of CAF (line 3 in Figure 3b). According to the literature, fullerenes can play several roles in photocatalytic systems: electron acceptor and mediator in reactions with solution species, as well as electron donor. In the latter case, the electron excited by light in the fullerene and transferred to the semiconductor can be involved in the photocatalytic process [23]. However, this option may be excluded because the absorption of light of the wavelength 400 nm by the fullerenes used in this work is very small (Figure 2c). On the other hand, the cyclic voltammograms of the fullerene derivatives used in this work indicated that the reduction potential $C_{60}X/C_{60}^{⦁-}X$ (where X is a functional group attached to the fullerene) is about −0.29 ± 0.02 V vs. SHE, being nearly independent of the type of substituent (*X*) (see Table S1 and an exemplary voltammogram in Figure S4b). This means that the LUMO level energy of the fullerenes is very close to the CB edge of BiVO_4_, and therefore, they do not help noticeably in the separation of the charge carriers photogenerated in BiVO_4_, and in consequence, in improving the CAF degradation.

In contrast, a substantial increase in the photodegradation rate of CAF with the use of BiVO_4_ was observed after the addition of Fe^3+^ ions (of concentration 50 ppm) to the studied solution (line 4 in Figure 3b). The standard redox potential of the Fe^3+^/Fe^2+^ couple is located at the potential of 0.77 V vs. SHE, i.e., below the CB of BiVO_4_ by about 1.1 V (see Figure 3a). Thus, the photogenerated electrons reduce Fe^3+^ ions, while the holes left in the valence band of BiVO_4_ may be efficiently involved in CAF oxidation.

### 2.3. Comparison of Photocatalytic Activity of BiVO_4_/g-C_3_N_4_ and BiVO_4_/Mediator/g-C_3_N_4_

**Figure 4:**
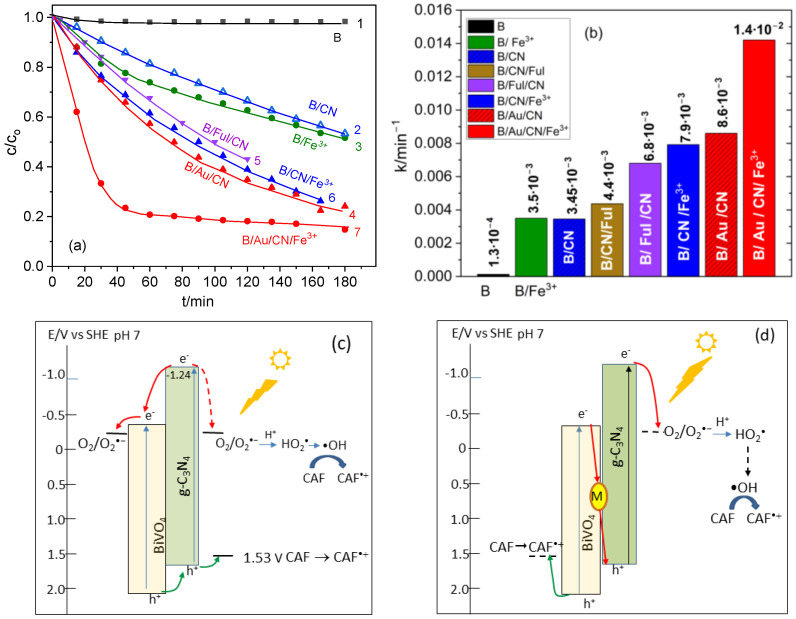
(**a**) Comparison of the plots c/c_o_ in a function of irradiation time (at diode 400 nm) of CAF solution in the presence of bare BiVO_4_ (B, line 1), BiVO_4_/g-C_3_N_4_ (B/CN, line 2), BiVO_4_/Fe^3+^ (B/Fe^3+^, line 3), BiVO_4_/Au/g-C_3_N_4_ (B/Au/CN, line 4), BiVO_4_/C_60_-MPB/g-C_3_N_4_ (B/Ful/CN, line 5), BiVO_4_/g-C_3_N_4_ in the presence of Fe^3+^ ions (B/CN/Fe^3+^, line 6), and BiVO_4_/Au/g-C_3_N_4_ in the presence of Fe^3+^ ions (B/Au/CN/Fe^3+^, line 7). (**b**) Comparison of apparent rate constants of the photocatalytic decomposition of CAF with the use of t different photocatalytic systems, obtained from the plots presented in (**a**). (**c**) Band diagram and proposed scheme of photocatalytic CAF degradation with the use of BiVO_4_/g-C_3_N_4_ without a mediator. (**d**) Band diagram and proposed scheme of photocatalytic CAF degradation with the use of BiVO_4_/M/g-C_3_N_4_, where M is the charge separation mediator: Au, fullerene, or Fe^3+^ ions.

The band diagram of g-C_3_N_4_ was constructed using the value of the valence band edge (1.57 V vs. SHE) determined by us from VB-XPS studies [38], and the band gap energy (2.82 eV) obtained from a diffuse reflectance spectrum (DRS) (Figure 2b). The obtained values are consistent with those reported in the literature [39,40,41]. Since the edges of the conduction and valence bands of g-C_3_N_4_ are located above those of BiVO_4_ (Figure 4c), an efficient electron–hole separation in the interfacial region should occur. Moreover, both semiconductors are photoactive at 400 nm, i.e., the number of charge carriers available in the photocatalytic process should be significantly higher compared to that in the single materials. However, as visible in Figure 4a, the rate of photocatalytic degradation of CAF in the presence of this hybrid system (abbreviated as B/CN) was very similar to that obtained for non-modified BiVO_4_ in the presence of Fe^3+^ ions in CAF solution (lines 2 and 3, respectively). The rate constants of the first stage of CAF degradation in both cases are about 3.5 · 10^−3^ min^−1^. These data suggest that the separation of photogenerated charge carriers occurs via the staircase mechanism, typical for a hybrid system of type II, rather than via Z-scheme. In the band diagram presented in Figure 4c, the upper edge of the valence band of g-C_3_N_4_ is located at the potential 1.57 V vs. SHE, which is very close to the onset potential of CAF oxidation. Thus, the holes injected from BiVO_4_ to g-C_3_N_4_ are less powerful in CAF oxidation than those generated in pure BiVO_4_. On the other hand, the lower edge of the CB of g-C_3_N_4_ is located at a much more negative potential (by about −0.92 V) than the CB edge of BiVO_4_. Therefore, the electrons from both semiconductors may be involved in the reduction of $O_{2}$ to $O_{2}^{⦁-}$, either directly (dashed line in the diagram) or by the mechanism typical for the type-II heterojunction (solid line), depending on the relative rates of the charge transfer at the semiconductor/solution and g-C_3_N_4_/BiVO_4_ interfaces.

To provide the recombination of less energetic electrons and holes generated in both semiconductors, three different charge transfer mediators were tested: Au NPs and fullerenes, deposited on BiVO_4_ before application of g-C_3_N_4_, and Fe^3+^ ions present in CAF solution. As visible in Figure 4a, the presence of the mediators led to a strong increase in the rate of CAF degradation, and the apparent rate constants increased by about twice the value obtained for the binary system BiVO_4_/g-C_3_N_4_ (Figure 4b).

It was interesting to note that the photocatalytic performance of the studied composites increased in the following order: BiVO_4_/C_60_-MPB/g-C_3_N_4_ < BiVO_4_/g-C_3_N_4_/Fe^3+^ < BiVO_4_/Au/g-C_3_N_4_. This may be explained by taking into account the relative positions of energy levels of the mediators with respect to the CB and VB edges of both semiconductors. As mentioned above, the LUMO potential of fullerenes is practically the same as the potential of the bottom edge of the CB of BiVO_4_. On the other hand, the fullerene core is considered as an excellent electron acceptor, and the interactions between π electrons of the fused aromatic rings and heptazine rings of g-C_3_N_4_ probably improve the adhesion between the two components of the hybrid system, facilitating the recombination of electrons from the CB of BiVO_4_ with holes from the VB of g-C_3_N_4_. It is worth noting that the systems with similar mediators containing the C_70_ core, instead of C_60_, revealed lower photocatalytic performance (Figure S6). This is probably a result of the lower solubility of C_70_ derivatives and their tendency to aggregate, despite slightly higher absorption in the visible light range (Figure 2d). In the case of the Fe^3+^ mediator, the redox potential of the Fe^3+^/Fe^2+^ couple is located just between the CB of BiVO_4_ and the VB of g-C_3_N_4_, and therefore, the electrons from BiVO_4_ reduce Fe^3+^ ions to Fe^2+^, while the holes from the VB of g-C_3_N_4_ oxidize Fe^2+^ back to Fe^3+^. However, the highly energetic electrons from the CB of g-C_3_N_4_ and the holes from the VB of BiVO_4_ could also be involved in these reactions, leading to some decrease in the efficiency of the photocatalytic process [42]. Moreover, the presence of Fe^3+^ ions slightly influences the UV-vis spectrum of the studied solution, since the absorption bands of aqueous complexes of Fe(III) ions and CAF are observed in a similar wavelength range (Figure S5b). The problems mentioned above do not exist in the case of the Au mediator, and therefore, the system BiVO_4_/Au/g-C_3_N_4_ revealed the highest photocatalytic activity in CAF degradation. The rate constant was about 2.5 times higher than that obtained in the presence of BiVO_4_/g-C_3_N_4_ without a mediator.

**Figure 5:**
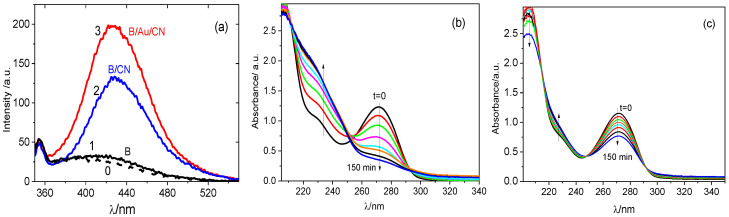
(**a**) PL spectra of the solution containing 5⋅10^−4^ M TA and 2⋅10^−3^ M NaOH after 10 min of illumination in the presence of BiVO_4_ (line 1), BiVO_4_/g-C_3_N_4_ (line 2), and BiVO_4_/Au/g-C_3_N_4_ (line 3). The dashed line (0) corresponds to the background solution (without illumination); the excitation wavelength was 315 nm. Evolution of UV-vis spectra of CAF solution during irradiation in the presence of (**b**) BiVO_4_/Au/g-C_3_N_4_ and (**c**) BiVO_4_/g-C_3_N_4_.

To compare the amount of hydroxyl radicals formed by the transformation of $O_{2}^{⦁-}$ with and without the Au mediator, the experiment with the use of terephthalic acid (TA) was performed. The PL spectra of the TA solutions irradiated for 10 min with a diode 400 nm, in the presence of BiVO_4_, BiVO_4_/g-C_3_N_4_, and BiVO_4_/Au/g-C_3_N_4_ photocatalysts, are presented in Figure 5a. The lack of a peak in the PL spectrum of the solution irradiated in the presence of pure BiVO_4_ confirms that no hydroxyl radicals were formed, probably because of very fast *e^−^-h^+^* recombination. In contrast, a well-developed peak at about 430 nm, related to TA-OH, appears in the spectra of the solutions irradiated in the presence of the hybrid systems, but its intensity is about 1.6 times higher when the Au mediator was used. This result may be an additional piece of evidence of different mechanisms of the photocatalytic process with and without the mediator. It was also interesting to note the differences in the UV-Vis spectra of CAF solutions, recorded during irradiation in the presence of BiVO_4_/g-C_3_N_4_ and BiVO_4_/Au/g-C_3_N_4_. As visible in Figure 5b, a strong decrease in the main absorption peak at 270 nm, observed in the presence of the photocatalyst with Au mediator, is accompanied by a substantial absorbance increase in the wavelength range 250–210 nm, with the formation of an isosbestic point at about 250 nm. The data in Figure 5c indicate that the changes in this wavelength range with the use of BiVO_4_/g-C_3_N_4_ are very small. Therefore, one can suppose that the system operating according to Z-scheme generates a higher amount of reactive oxygen species ($O_{2}^{•-}$ and then •OH radicals), which together with more oxidative holes lead to more efficient CAF degradation, and concentrations of degradation products which absorb in the UV range are much higher than those found without Au mediator. However, in order to obtain the quantitative results and specify the photocatalytic degradation products, additional measurements with the use of HPLC and LC-MS methods should be carried out.

The crucial role of the superoxide radicals in the whole photocatalytic process was confirmed by very strong hindering of CAF degradation after deaeration of the solution (compare Figure S5c,d). Moreover, deoxygenation of the TA solution led to a significant decrease in TA-OH formation under irradiation of BiVO_4_/Au/g-C_3_N_4_ photocatalyst, confirming that the hydroxyl radicals are formed by transformation of superoxide radicals (via Reactions (1)–(3)).

It was very interesting to note that an extremely high degradation rate of CAF was obtained with the use of BiVO_4_/Au/g-C_3_N_4_ system irradiated in the presence of Fe^3+^ ions in the solution (line 7 in Figure 4a). This may be explained by a synergic effect of the two mediators but also by the Fenton reaction, which likely occurs in this system. In the latter case, the Au nanoparticles act as the Z-scheme mediator, while the H_2_O_2_ formed in Reaction (2) can react with Fe^2+^ obtained by reduction of Fe^3+^ ions, according to the scheme:

(4) $${Fe}^{2+}+H_{2}O_{2}\to {Fe}^{3+}+•OH+{OH}^{-}$$

The rate constant of this reaction is very high (40–80 M^−1^s^−1^ [43]), and an additional amount of hydroxyl radicals may be involved in the first step of CAF degradation.

**Figure 6:**
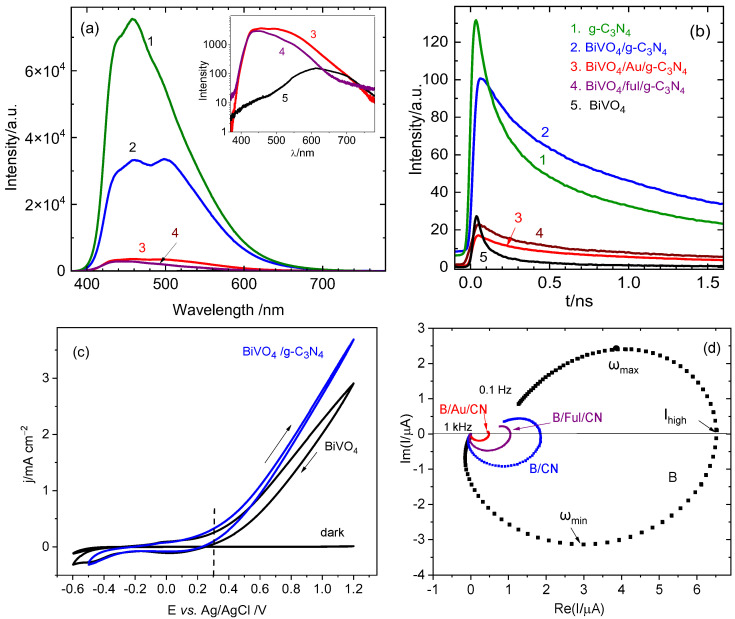
(**a**) Photoluminescence spectra and (**b**) PL transients recorded at 530 nm in air for g-C_3_N_4_ (curves1), BiVO_4_/g-C_3_N_4_ (curves 2), BiVO_4_/Au/g-C_3_N_4_ (curves 3), BiVO_4_/C_60_-MPhB/g-C_3_N_4_ (curves 4) and pure BiVO_4_ (curves 5) at optical excitation wavelength 300 nm; inset: comparison of PL data in semi-logarithmic scale for better comparison of the results. (**c**) Cyclic voltammograms of FTO/BiVO_4_ and FTO/BiVO_4_/g-C_3_N_4_ electrodes in the solution of 0.1 M Na_2_SO_4_ in dark and under illumination with a diode 365 nm at illumination intensity 100 mW cm^−2^. (**d**) Comparison of IMPS spectra for BiVO_4_, BiVO_4_/Au (B/Au), BiVO_4_/g-C_3_N_4_ (B/CN), BiVO_4_/fullerene/g-C_3_N_4_ (B/Ful/CN), and BiVO_4_/Au/g-C_3_N_4_ (B/Au/CN), obtained in aqueous solution of 0.1 M Na_2_SO_4_ at the polarization potential 0.3 V vs. Ag/AgCl at the wavelength 370 nm (frequency range from 0.1 Hz to 1 kHz).

To compare the dynamics of the recombination process in the pristine components BiVO_4_, g-C_3_N_4_ and three hybrid systems BiVO_4_/g-C_3_N_4_, BiVO_4_/Au/g-C_3_N_4_, and BiVO_4_/fullerene/g-C_3_N_4_, photoluminescence measurements were performed. As visible in Figure 6a, the strongest emission band with a maximum at about 458 nm (2.71 eV), with a small shoulder at about 440 nm (2.82 eV), was obtained for pure g-C_3_N_4_ (line 1). Similar spectra have been reported in the literature, and these two bands were attributed to π*→LP (lone pair) and σ*→LP transitions, respectively [44,45]. The lone pair state of the nitrogen atom is a part of the valence band, while the excited states σ* and π*, consisting of sp^3^ C-N and sp^2^ C-N bonds, are located in the conduction band. The energy corresponding to these transitions fits well with the band gap energy of g-C_3_N_4_. In contrast, the PL spectrum of pristine BiVO_4_ has the lowest intensity, and it consists of two very broad overlapping bands: the higher one at about 605 nm (2.05 eV) and the smaller at about 490 nm (2.53 eV), visible as a hump (line 5 in the inset in Figure 6a). The second value is quite close to the band gap energy of BiVO_4_ determined from the absorption edge in the UV-vis spectrum (2.43 eV), while a broad maximum at 600 nm, also reported by other authors, suggests the recombination via intermediate electronic states located within the band gap [46]. Very weak photoluminescence of BiVO_4_ seems to be in contradiction with the high efficiency of electron–hole recombination in this material, postulated in the literature [47,48], and this discrepancy can be explained by non-radiative recombination. According to Abdellaoui et al., the recombination of photocarriers in BiVO_4_ occurs predominantly through multiphonon nonradiative recombination via deep level defects, making short lifetimes of few nanoseconds [49]. As visible in Figure 6a, the deposition of g-C_3_N_4_ on the surface of BiVO_4_ resulted in a significant quenching of PL of g-C_3_N_4_ (about 2.5 times), while after incorporation of Au NPs or fullerene (C_60_-MPhB) mediators between these two semiconductors, the PL intensity decreased 20 times in comparison to that recorded for g-C_3_N_4_ alone and about 10 times with respect to PL intensity of BiVO_4_/g-C_3_N_4_ (lines 3 and 4). This may be ascribed to strong improvement of the charge carrier separation in the ternary system with respect to that in the binary one, and may be explained again by different mechanisms of the charge transfer, i.e., staggered for BiVO_4_/g-C_3_N_4_ and Z-scheme in the presence of the mediator. The time-resolved photoluminescence spectra presented in Figure 6b provide information on the dynamics of the charge transfer. As visible, the photoluminescence of BiVO_4_ decays very rapidly within the first 0.4 ns but a small PL tail, well visible in the semi-logarithmic plot (Figure S7, line 5), extends up to 1.5 ns. The presence of these two segments with lifetimes of 0.045 ns and 1.93 ns, respectively, suggests very fast electron–hole recombination just after excitation, followed by much slower annihilation of the charge carriers which travel through the semiconductor and need more time to recombine. A very fast decrease in PL signal is also observed for pure g-C_3_N_4_ deposited on the FTO substrate (curve 1 in Figure 6b). The transient is also composed of two segments with two different lifetimes, τ_1_ = 0.14 ns and τ_2_ = 1.63 ns.

After deposition of g-C_3_N_4_ on BiVO_4_, an initial decay of the PL signal was slowed down (the lifetime corresponding to this segment increased twice), while the second part has a slope similar to that for g-C_3_N_4_. This difference in the slopes of the initial parts of the transients can be explained by the injection of highly mobile electrons from g-C_3_N_4_ to BiVO_4_. In the case of BiVO_4_/Au/g-C_3_N_4_ and BiVO_4_/C_60_-MPhB/g-C_3_N_4_ systems, the emission is strongly suppressed due to non-radiative recombination of the electrons from BiVO_4_ with the holes from g-C_3_N_4_ via the mediators.

In order to investigate the influence of the solid mediator on the dynamics of the charge transfer at the photocatalyst/solution interface and electron–hole surface recombination process, the measurements with the use of intensity-modulated photocurrent spectroscopy (IMPS) were performed. IMPS is a powerful method developed by Peter et al. [50,51,52] for determining the rate constants of the charge transfer (*k_tr_*) and surface recombination (*k_r_*). For example, by the use of this method, it has been shown that the photocurrent of BiVO_4_ is not limited by water oxidation kinetics but by surface recombination, which was diminished by deposition of Co-based catalyst on BiVO_4_ [48]. We performed the IMPS measurements for bare BiVO_4_, BiVO_4_/g-C_3_N_4_, BiVO_4_/fullerene/g-C_3_N_4_, and BiVO_4_/Au/g-C_3_N_4_ photocatalysts in aqueous solution of 0.1 M Na_2_SO_4_ under modulated illumination at the wavelength of 370 nm, at a constant potential of 0.3 V vs. Ag/AgCl. The selected potential is close to the photocurrent onset potential (dashed line in Figure 6c) to minimize the influence of external electrode polarization on the separation of charge carriers. The course of the obtained IMPS plots, presented in Figure 6d, is consistent with the response predicted theoretically by Peter et al. [50,52]. According to their model, the high- frequency loop (in the fourth quadrant) is associated to the cell time constant, while the low-frequency semicircle (“recombination semicircle” in the upper quadrant) reflects the competition between charge transfer and surface recombination, according to the equation [48]:

(5) $$\omega_{max}=k_{tr}+k_{rec}$$

where $\omega_{max}=2\pi f_{max}$ is the radial frequency corresponding to the maximum of the upper semicircle.

On the other hand, the ratio *k_tr_*/(*k_tr_ + k_rec_*) can be determined using the relationship [51]:

(6) $$\frac{I_{low}}{I_{high}}=\frac{k_{tr}}{k_{tr}+k_{rec}}$$

where *I_low_* and *I_high_* are low- and high-frequency intersections of the semicircle with the real axis. The current intersection at low frequency corresponds to steady state photocurrent in *i-t* response, since it is related to the hole transfer at the interface under steady state conditions, while *I_high_* represents an instantaneous photocurrent which is strongly affected by recombination [53]. As visible in Figure 6d, the highest value of *I_high_* was obtained for bare BiVO_4_, while the combination of BiVO_4_ with g-C_3_N_4_ and the formation of the ternary systems BiVO_4_/fullerene/g-C_3_N_4_ and BiVO_4_/Au/g-C_3_N_4_ led to a significant and successive diminution of the semicircles’ radii, with a concomitant decrease in the value of *I_high_*. Moreover, the upper semicircle is less developed for BiVO_4_/fullerene/g-C_3_N_4_, and finally, it is not formed for the BiVO_4_/Au/g-C_3_N_4_ system. A disappearance of the upper loop, reported in the literature by Rodriguez-Gutiérrez et al. for the WO_3_/BiVO_4_ heterojunction, has been explained by extraction of photogenerated electrons by WO_3_, preventing the surface recombination [54]. We ascribe the similar behavior observed for BiVO_4_/Au/g-C_3_N_4_ to the improved charge separation at the heterojunction due to the Z-scheme mechanism.

## 3. Materials and Methods

### 3.1. Materials

All reagents were of analytical grade and used without further purification. Bismuth nitrate (Bi(NO_3_)_3_, 98%), vanadyl acetylacetonate (VO(acac)_2_, 98%), dimethyl sulfoxide (DMSO, 99.9%), caffeine (1,3,7-trimethylxantine, CAF), terephthalic acid (TA), tetrabutylammonium hexafluorophosphate (TBAPF_6_), chloroauric acid (HAuCl_4_) and Nafion^®^ 117 solution were purchased from Sigma-Aldrich (Darmastadt, Germany). Sodium hydroxide (NaOH, 99.8%), sodium sulfate Na_2_SO_4_, iron (III) nitrate Fe(NO_3_)_3_, urea, absolute ethanol and HNO_3_ (70%) were purchased from POCh S.A. (Gliwice, Poland), while *p*-benzoquinone (98%) was provided by FLUKA (Buchs, Switzerland).

The aqueous solutions were prepared using deionized (DI) water (Rephile, resistivity 18 MΩ cm). A conducting FTO (F-doped tin oxide) glass of a resistance of 20 Ω square^−1^ was obtained from Dyenamo AB (Stockholm, Sweden).

### 3.2. Deposition of BiVO_4_ on FTO

BiVO_4_ was formed on an FTO substrate according to a two-step procedure reported in the literature [55,56]. The FTO plates (of the size 2.5 × 1 cm) were cleaned and activated according to the procedure described elsewhere [30]. In the first step, FTO was covered with BiOI by electrodeposition performed in the solution containing [BiI_4_]^−^ complex and *p*-benzoquinone, in a typical three-electrode cell with Ag/AgCl, Cl^−^ (3 M KCl) as the reference electrode, and a Pt plate as the counter electrode, as reported in the literature. The FTO electrode was cycled (40 cycles) in the potential range from 0.33 V to −0.25 V at the scan rate of 50 mV s^−1^ to achieve the overall charge density of 380 mC cm^−2^ [30]. Then, the layer of deep orange BiOI was transformed into yellow BiVO_4_ by reaction with vanadium precursor, VO(acac)_2_ dissolved in DMSO, performed at a temperature 450 °C, for 2 h, according to the procedure reported in the literature [39].

### 3.3. Deposition of Au Nanoparticles and Fullerene Derivatives on the Surface of BiVO_4_

Au nanoparticles (Au NPs) were obtained by two different methods. In the first one, an aqueous solution of HAuCl_4_ of concentration 9 mM was applied by a drop casting on the surface of BiVO_4_ deposited on FTO (100 μL applied on the surface area of 2 cm^2^). The samples were dried on a hot plate at a temperature of 35 °C, and then annealed in an oven at 400 °C for 1 h. Au nanoparticles obtained by this method are abbreviated as Au-d. In the second method, a thin layer of gold was deposited on BiVO_4_ by sputtering with the use of Emitech K575X Sputter Coater (East Sussex, UK). Then, the sample was annealed in an oven at a temperature of 400 °C for 1 h to convert the film into Au nanoparticles (Au-s).

The fullerene derivatives with C_60_ and C_70_ cores, 61-Bis(ethyloxycarbonyl)-1,2-methano[60]fullerene (C_60_-MEB), 61-(Ethyloxycarbonyl)-61-(benzyloxycarbonyl)-1,2-methano[60]fullerene (C_60_-MPhB), and 61-(Ethyloxycarbonyl)-61-(pyren-1-yl-methyloxy-carbonyl)-1,2-methano[60]fullerene (C_60_-MPB), and their C_70_ analogues, presented in the inset in Figure 2d, were synthesized according to the procedures described elsewhere [57].

After synthesis, the fullerene derivatives were dissolved in dichloromethane (DCM) by sonication for 30 min. Then methanol was added with 1:1 vol. ratio, and the mixture was sonicated for another 2 h. Finally, the solvent was evaporated at 40 °C. The obtained powders (6 mg) were dissolved in the mixture containing 600 μL of H_2_O (DI), 175 μL of ethanol and 25 μL of Nafion with the use of ultrasonic bath (1 h). The obtained “inks” of a volume of 10 μL were applied by drop casting on the surface of BiVO_4_ deposited on FTO and the samples were dried for 1h at temperature 125 °C.

The energies of HOMO and LUMO levels of C_60_ and C_70_ derivatives were determined from the oxidation and reduction onset potentials in the cyclic voltammograms recorded on the Pt electrode in dichloromethane (DCM) containing the fullerene derivatives and 0.1 M TBAPF_6_ supporting electrolyte [57]. The band gap energies of all fullerene derivatives studied in this work were determined from the difference E_LUMO_-E_HOMO_ and from UV-Vis absorption spectra. The obtained data, presented in Table S1 (in Supplementary Materials), indicate that attachment of single or fused rings to the malonate group does not influence the positions of HOMO and LUMO levels as well as on the band gap energy of the fullerene.

### 3.4. Preparation of BiVO_4_/g-C_3_N_4_, BiVO_4_/Au(NPs)/g-C_3_N_4_ and BiVO_4_/Fullerene/g-C_3_N_4_ Systems

Graphitic carbon nitride was synthesized by thermal condensation of urea, according to the procedure described elsewhere [58]. The obtained powder was exfoliated in DMSO (30 mg in 25 mL) in an ultrasonic bath (120 W) for 8 h, and the suspension was centrifuged at 3000 rpm for 10 min to remove the non-exfoliated polymer. The exfoliated g-C_3_N_4_ in the form of thin lamellas was applied by drop casting in 3 portions (150 μL each) on the surfaces of FTO, FTO/BiVO_4_, FTO/BiVO_4_/Au(NPs) and FTO/BiVO_4_/fullerene, placed on the hotplate (at 125 °C). After application of the last portion, the samples were kept for 1 h, at 200 °C.

### 3.5. Characterization Methods

A field-emission scanning electron microscope (FE-SEM) (Merlin, Carl Zeiss, Oberkochen, Germany) at an operating voltage of 3 kV was used to examine the morphology of the samples. The composition of the films deposited on the surface of FTO plates was determined from EDS elemental analysis (at an accelerating voltage of 15 kV).

Diffuse reflectance spectra (DRS) of the samples were recorded using a UV-Vis spectrometer (Shimadzu UV-3600, Kyoto, Japan) equipped with an integrating sphere. The same spectrophotometer or Lambda 12 (PerkinElmer, Shelton, CT, USA), working in a transmission mode in the wavelength range 1100–190 nm, were used to monitor the concentration changes of CAF due to adsorption and photocatalytic degradation.

FTIR spectra were recorded with the use of Nicolet iS 50 FTIR spectrometer (Thermo Fisher Scientific, Waltham, MA, USA), in the reflection mode in the wavenumber range 4000–400 cm^−1^.

The photoluminescence (PL) measurements were performed using Ti:sapphire pulsed laser (80 MHz frequency) (Coherent, Santa Clara, CA, USA) at the wavelength 300 nm for optical excitation. The spectral and temporal distribution of the PL was analyzed by a 300 mm monochromator and a streak camera (Hamamatsu, Japan) of about 3 ps time resolution. The measurements were performed at room temperature. All samples were deposited on FTO substrate. The control experiment performed for g-C_3_N_4_ applied on the glass has shown that the FTO substrate does not improve the separation of the photogenerated charge carriers.

Electrochemical measurements were performed with Autolab PGSTAT302N (Metrohm, Utrecht, The Netherlands) in a Teflon electrochemical cell with a quartz window. The working electrode was FTO-covered with the photocatalyst, a Pt wire was the counter electrode and Ag/AgCl/Cl^−^ (3 M KCl) was the reference electrode. Electrochemical measurements were performed in 0.1 M Na_2_SO_4_ supporting electrolyte. The potential was recalculated to the standard hydrogen electrode (SHE) using the equation:

(7) $$E\left(V,\mathrm{vs}.\;SHE\right)=E\left(V,\mathrm{vs}.\;Ag/AgCl\right)+0.21\;V$$

### 3.6. Photocatalytic Tests and Detection of •OH Radicals

The FTO plates covered with the photocatalysts were placed in a special Teflon holder and immersed in a quartz cuvette containing caffeine (CAF) as the model pollutant, at a concentration of 10 ppm. Prior to photocatalytic measurements, the samples were conditioned in the dark in the pollutant solution to achieve adsorption/desorption equilibrium. Then, the samples were irradiated with a high-power LED (400 nm) with continuous magnetic stirring of the solution. The power density of illumination in the place of the sample, measured with IL1700 Radiometer (International Light Technologies, Peabody, MA, USA), was 100 mW cm^−2^.

Degradation of CAF with an initial concentration of 10 ppm, under irradiation in the presence of the photocatalysts, was monitored by recording the UV-Vis spectra of the solutions in 5–30 min periods, depending on the sample. In the UV-Vis spectrum of CAF, two characteristic bands develop, with maxima at about 270 and 205 nm. Since the second peak is located close to the measurement limit of the instrument (190 nm), the changes in the intensity of the peak at 270 nm were taken to determine the changes in CAF concentration during the photocatalytic process. The CAF degradation rates with the use of different systems were compared by plotting *c/c_o_* as a function of time, where *c_o_* is an initial concentration, while *c* is the concentration at time *t*. The photocatalytic degradation rate constant was determined using the Langmuir–Hinshelwood model, according to which the equation for the reaction rate (*r*) for diluted solutions is simplified to a pseudo-first-order kinetic law: $r=k_{app}c$, where *k_app_* is an apparent rate constant.

The formation of the hydroxyl radicals during the photocatalytic process was monitored by the conversion of terephthalic acid (TA) into highly fluorescent 2-hydroxy-terephthalic acid (TA-OH) [58], according to the procedure reported elsewhere [30].

### 3.7. IMPS Measurements

The IMPS (intensity-modulated photocurrent spectroscopy) measurements were carried out with the use of a potentiostat equipped with IMPS module (Instytut Fotonowy, Kraków, Poland), in a three-electrode electrochemical cell with Ag/AgCl/Cl^−^ reference electrode and Pt counter electrode, in a solution of 0.1 M Na_2_SO_4_. The light source was an LED at a wavelength of 370 nm, calibrated via a silicon-based radiometer (Instytut Fotonowy, Kraków, Poland). The light was sinusoidal modulated in the frequency range 1 kHz–0.1 Hz. Before measurements, the working electrodes (FTO/photocatalyst) were tested by CV measurements in the dark and under a 365 nm diode to test their photoelectrochemical activity and select the most appropriate potential for IMPS measurement.

## 4. Conclusions

In this work, we proved that separation of photoinduced electron–hole pairs at the BiVO_4_/g-C_3_N_4_ interface occurs via the mechanism typical of heterojunction of type II, with the electron transfer from the CB of g-C_3_N_4_ to the CB of BiVO_4_, and the holes between the VBs of both semiconductors in the opposite direction. After incorporation of the mediator between BiVO_4_ and g-C_3_N_4_, the rate constant of photocatalytic degradation of CAF increased two times due to the change of the charge carrier separation mechanism from the staggered to the Z-scheme. The improved e-h separation was confirmed by PL, TRPL, and IMPS measurements. We have shown for the first time that C_60_ fullerene derivatives can play the role of the charge separation mediator in this system, despite the LUMO energy level being located just below the CB edge of BiVO_4_. It was shown that the system with the Au mediator reveals higher photocatalytic activity than that with the fullerene or Fe^3+^/Fe^2+^ shuttle mediator. An extremely strong increase in the CAF degradation rate was obtained with the system BiVO_4_/Au/g-C_3_N_4_ irradiated in the presence of Fe^3+^ ions in CAF solution. This improvement is not only due to the synergic effect of the two mediators but also a result of the Fenton reaction, which probably occurs in this system.

## Figures and Tables

**Table 1:** Results of elemental analysis of the samples by EDX.

Element	At%			
	BiVO_4_/Au-d	BiVO_4_/Au-s	BiVO_4_/Au-d/g-C_3_N_4_	BiVO_4_/Fullerene
Au	2.11	5.87	1.52	-
Bi	27.96	22.56	13.03	16.79
V	22.43	26.86	12.73	17.65
O	41.12	36.86	13.55	33.40
C	-	-	25.26	20.91
N	-	-	32.67	-

## Data Availability

Data available on request due to privacy restrictions.

## Supplementary Materials

The following supporting information can be downloaded at: https://www.mdpi.com/article/10.3390/molecules29215092/s1, Figure S1: SEM image of BiVO_4_/Au(layer) and TEM image of exfoliated g-C_3_N_4_; Figure S2: Tauc’s plots for BiVO_4_, g-C_3_N_4_ and BiVO_4_/g-C_3_N_4_ composites; Figure S3: Cyclic voltammogram of the CAF solution and C_60_-MPhB solution; Figure S4: Evolution of UV-Vis spectra during CAF photocatalytic degradation in the presence of different photocatalysts; Figure S5: Plots of c/c_o_ vs. time for CAF photocatalytic degradation in the presence of BiVO_4_/C_60/70_-MPB/g-C_3_N_4_; Figure S6: Semi-logarithmic plot of PL transients in air in the presence of different composites; Scheme S1: Oxidation of CAF by the holes; Scheme S2: Oxidation of CAF by hydroxyl radicals; Table S1: HOMO and LUMO energies of fullerene derivatives.
