# Supplementary material for: Z-Scheme BiVO4/g-C3N4 Photocatalyst—With or Without an Electron Mediator?
Source: Molecules. 2024 Oct 28;29(21):5092. doi: 10.3390/molecules29215092 (PMC11547383; doi:10.3390/molecules29215092)
Supplement: Supplementary file 1 [file molecules-29-05092-s001.zip › molecules-3236185-supplementary.pdf]

Supplementary Materials

Z-scheme $BiVO_4$/g-C_3N_4 photocatalyst – with or without electron mediator?

Tomasz Łęcki, ‡[a,b]*, Kamila Zarębska,* ‡[a,b] *Ewelina Wierzyńska*[a]*, Krzysztof P. Korona*[c]
Paulina Chyży [a]*, Piotr Piotrowski* [a] *and Magdalena Skompska* [a,b*]

a) Laboratory of Electrochemistry, Faculty of Chemistry, University of Warsaw, Pasteur 1, 02-093 Warsaw, Poland
b) Biological and Chemical Research Centre, Faculty of Chemistry, University of Warsaw, Żwirki i Wigury 101, 02-089 Warsaw, Poland
c) Faculty of Physics, University of Warsaw, Pasteur 5, 02-093 Warsaw, Poland

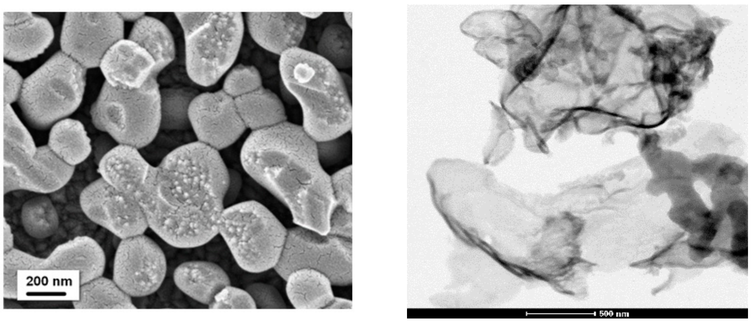

(a) (b)

Figure S1. SEM image of $BiVO_4$/Au (layer) obtained by Au sputtering on $BiVO_4$ deposited on FTO (a) and TEM image of exfoliated g-C_3N_4 (b).

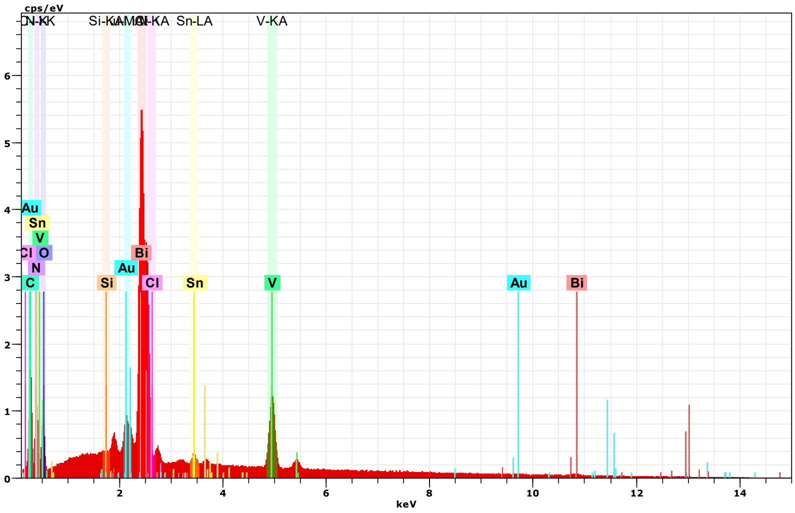

Figure S2. EDX spectrum of $BiVO_4$/Au(NPs)/g-C_3N_4 deposited on FTO

* Corresponding author: Magdalena Skompska, Faculty of Chemistry, University of Warsaw, Pasteur 1, Warsaw, Poland; e-mail: mskomps@chem.uw.edu.pl
‡ These authors contributed equally

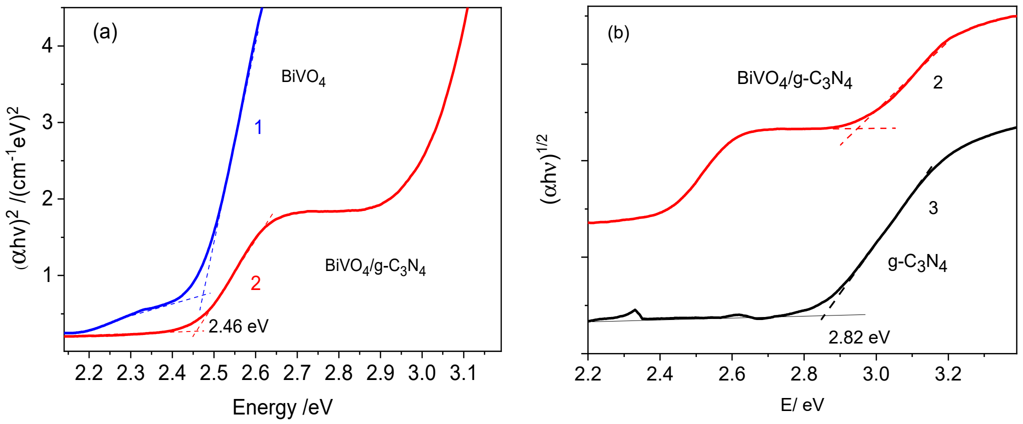

Figure S3. Tauc's plots for $BiVO_4$ (line 1) and $BiVO_4/g\text{-}C_3N_4$ (line 2) (a), and for $g\text{-}C_3N_4$ (line 3) and $BiVO_4/g\text{-}C_3N_4$ (line 2) (b), taking into account that $BiVO_4$ is direct semiconductor, while $g\text{-}C_3N_4$ is indirect semiconductor.

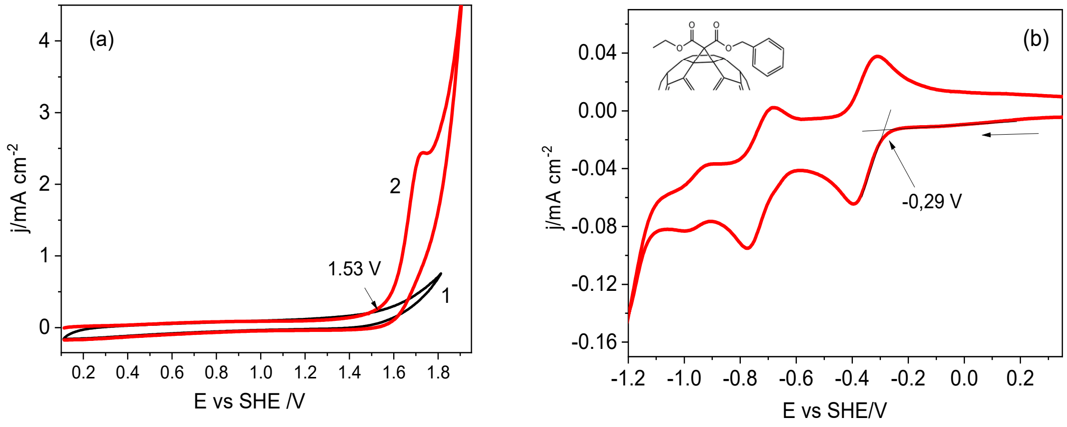

Figure S4. a) Cyclic voltammograms on GC electrode in the solution of 0.1 M Na_2SO_4 (line 1) and in 0.1 M Na_2SO_4 containing 0.012 M CAF (line 2),

b) Cyclic voltammogram on GC electrode in DCM solution containing C_{60}-MPhB and $TBAPF_6$ as the supporting electrolyte.

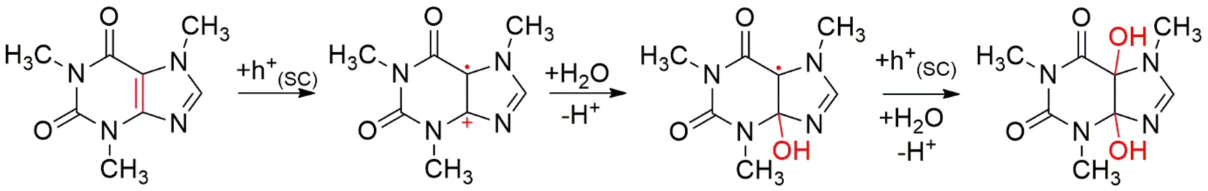

Scheme S1. Oxidation of CAF by the holes photogenerated in the valence band of semiconductor (SC) [1].

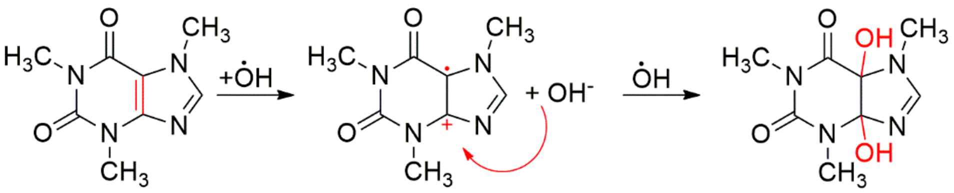

Scheme S2. Oxidation of CAF by the hydroxyl radicals [1].

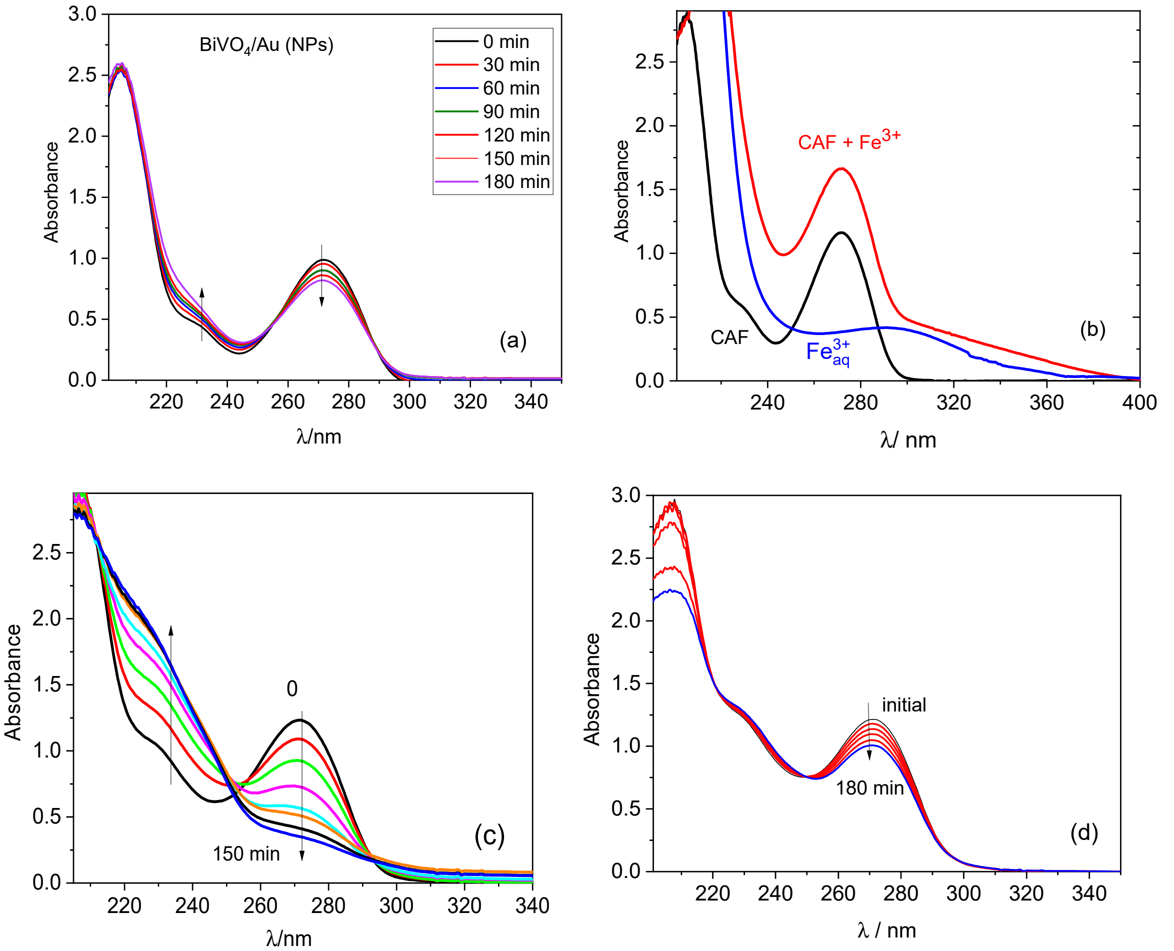

Figure S5. a) Evolution of The UV-Vis spectra of CAF solution under illumination with diode at 400 nm in the presence of $BiVO_4$/Au (NPs),

b) comparison of UV-Vis spectra of aqueous the solutions containing: CAF, $FeCl_3$ and the mixture of both compounds,

c) and d) Evolution of The UV-Vis spectra of CAF solution under illumination with diode at 400 nm in the presence of $BiVO_4$/Au(NPs)/g-C_3N_4 in the presence of oxygen (c) and after deaeration by bubbling of Ar (d).

Table S1. Potentials of HOMO and LUMO levels with respect to standard hydrogen electrode (SHE) and bandgap energies of the investigated fullerene derivatives

Fullerene derivative	HOMO *vs* SHE /V	LUMO *vs* SHE/V	$E_{g(el)}$/eV	$E_{g(opt)}$/eV	ref
C_{60}-MEB	1.59	-0.31	1.90	1.91	[2]
C_{60}-MPhB	1.58	-0.28	1.86	1.91	
C_{60}-MPB	1.57	-0.27	1.84	1.74	
C_{70}-MEB	1.57	-0.27	1.84	1.80	
C_{70}-MPhB	1.51	-0.30	1.81	1.80	
C_{70}-MPB	1.55	-0.27	1.82	1.78	

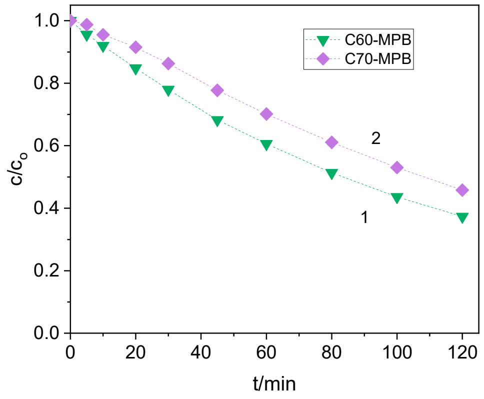

Figure S6. Comparison of the plots c/c$_o$ in a function of irradiation time (at diode 400 nm) of the CAF solution in the presence of $BiVO_4/C_{60}$-MPB/g-C_3N_4 (line 1) and $BiVO_4/C_{70}$-MPB/g-C_3N_4 (line 2).

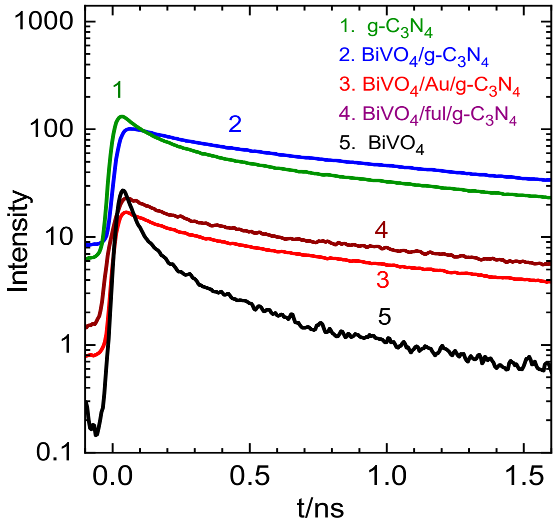

Figure S7. Semi-logarithmic plot of PL transients at 530 nm recorded in air for of g-C_3N_4 (line1), $BiVO_4$/g-C_3N_4 (line 2), $BiVO_4$/Au/g-C_3N_4 (line 3), $BiVO_4/C_{60}$-MPh/g-C_3N_4 (line 4) and pure $BiVO_4$ (line 5).

References

1. Łęcki, T.; Hamad, H.; Zarębska, K.; Wierzyńska, E.; Skompska, M. Mechanistic Insight into Photochemical and Photoelectrochemical Degradation of Organic Pollutants with the Use of $BiVO_4$ and $BiVO_4$/Co-Pi. *Electrochim. Acta* **2022**, *434*, 141292, doi:10.1016/j.electacta.2022.141292.

2. Mech, W.; Piotrowski, P.; Zarębska, K.; Korona, K.P.; Kaminska, M.; Skompska, M.; Kaim, A. The Impact of the Presence of Aromatic Rings in the Substituent on the Performance of C60/C70 Fullerene-Based Acceptor Materials in Photovoltaic Cells. *J. Electron. Mater.* **2022**, *51*, 6995–7008, doi:10.1007/s11664-022-09929-5.
